# Shift from a Zero-COVID strategy to a New-normal strategy for controlling SARS-COV-2 infections in Vietnam

**DOI:** 10.1017/S0950268823001048

**Published:** 2023-07-04

**Authors:** Do Thi Thanh Toan, Thanh Hai Pham, Khanh Cong Nguyen, Quang Thai Pham, Duc Doanh Ha, Hoa L. Nguyen, Robert J. Goldberg, Loc Quang Pham, Giang Minh Le, Tu Khac Nguyen, Van Khanh Tran, Van Thanh Ta

**Affiliations:** 1 School of Preventive Medicine and Public Health, Hanoi Medical University, Hanoi, Vietnam; 2 National Institute of Hygiene and Epidemiology, Hanoi, Vietnam; 3 Department of Population and Quantitative Health Sciences, University of Massachusetts Chan Medical School, Worcester, Massachusetts, USA; 4 Bac Ninh Center for Disease Control and Prevention, Bac Ninh, Vietnam; 5 Hanoi Medical University, Hanoi, Vietnam

**Keywords:** new-normal, SARS-COV-2, strategy, Vietnam, zero-COVID

## Abstract

The aim of this study is to analyse the changing patterns in the transmission of COVID-19 in relation to changes in Vietnamese governmental policies, based on epidemiological data and policy actions in a large Vietnamese province, Bac Ninh, in 2021. Data on confirmed cases from January to December 2021 were collected, together with policy documents. There were three distinct periods of the COVID-19 pandemic in Bac Ninh province during 2021. During the first period, referred to as the ‘Zero-COVID’ period (01/04–07/04/2021), there was a low population vaccination rate, with less than 25% of the population receiving its first vaccine dose. Measures implemented during this period focused on domestic movement restrictions, mask mandates, and screening efforts to control the spread of the virus. The subsequent period, referred to as the ‘Transition’ period (07/05–10/22/2021), witnessed a significant increase in population vaccination coverage, with 80% of the population receiving their first vaccine dose. During this period, several days passed without any reported COVID-19 cases in the community. The local government implemented measures to manage domestic actions and reduce the time spent in quarantine, and encouraged home quarantining for the close contacts of cases with COVID-19. Finally, the ‘New-normal’ stage (10/23–12/31/2021), during which the population vaccination coverage with a second vaccine dose increased to 70%, and most of the mandates for the prevention and control of COVID-19 were reduced. In conclusion, this study highlights the importance of governmental policies in managing and controlling the transmission of COVID-19 and provides insights for developing realistic and context-specific strategies in similar settings.

## Background

Early during the COVID-19 pandemic, countries that used a Zero-COVID strategy, which are non-pharmacological interventions that are designed to eliminate community transmission of this virus [1], were often the most successful in avoiding and/or minimising the deleterious effects of this ongoing pandemic [2]. In contrast, the New-normal strategy, which entails an adaptation process while maintaining preventive approaches [3], could reduce the impact of the COVID-19 pandemic on life’s daily activities once the pandemic is over [3].

The Vietnamese National Committee for Prevention and Control against COVID-19 reported that 2021 COVID-19 had caused many practical and financial hardships to residents living in the major cities of Vietnam and several large provinces. While many provinces, including Bac Ninh province, witnessed a successful shift from the Zero-COVID to the New-normal strategy [4], many knowledge gaps remain about how to best shift between these two strategies during a pandemic. Vietnam had successfully controlled the community spread of COVID-19 by applying the Zero-COVID strategy approach in 2020 [5, 6], and the government subsequently transitioned from this strategy to a New-normal strategy in 2021 to maintain the economic system of the country and local areas.

We analysed trends in the COVID-19 pandemic in relation to changes in governmental policies, based on epidemiologic data and policy actions in Bac Ninh, one of the largest provinces of Vietnam, during the 2021 wave.

## Methods

### Study setting

Bac Ninh province is in the Red River Delta region of northwestern Vietnam, with a population of 1,368,840 in 2019 [7]. It has eight districts with many concentrated industrial and information technology parks [8]. The occupational landscape of this province in 2019 was composed of the following: the agricultural, forestry, and fishery sectors accounted for 16%; the industrial and construction sectors accounted for 52%; while nearly one-third of all residents in the Bac Ninh province were part of the services sector [9].

### Study definitions

#### Control strategy working definitions

Following the WHO classification of COVID-19 control measures as policy actions for the control of the COVID-19 pandemic [10], we separated the policy actions in Bac Ninh province, in accordance with the national strategy of Vietnam, into six action groups: domestic movement, mask mandate, measurement and classification, prevention and controlling, and screening and vaccination [11–13] (Supplementary Appendix Table 1).

#### Study periods

We split the time span of the pandemic in Bac Ninh province into three periods using the following working definitions. The first period, the Zero-COVID period, went from 1 January to 4 July 2021, and its primary goal was to stop the community transmission of COVID-19. The next period, the Transition period, was from 5 July to 22 October 2021. During this period, the goal was to control community transmission of COVID-19 and minimise the importation of new cases from affected provinces while increasing the population level of COVID-19 vaccine coverage. The final period, the New-normal, was carried out from 23 October until the end of the study on 31 December 2021. During this period, the primary goal was to open social facilities and terminate all isolation policies.

#### COVID-19 containment cycle

We defined the COVID-19 containment cycle (CCC) as a set of non-pharmacological control measures to control the COVID-19 pandemic in each community in the Bac Ninh province. The CCC started with the detection of index cases of COVID-19 that were diagnosed in the community, which was followed by the implementation of several public health measures to control the transmission of SARS-Cov-2.

The CCC ended 14 days after the last cases of community transmission were detected. Following the guidelines of the Vietnam National Steering Committee, we used 14 days as the cut-off point for calculating the CCC. This time was calculated between the first day of community transmission (having more than one case of COVID-19 in the community) and the ending date, which was defined as occurring after 14 days with no COVID-19 cases being diagnosed in the community [11].

### Study design

We conducted an ecological study based on existing data. We collected data from the Bac Ninh province Center for Disease Control (CDC) from 1 January to 31 December 2021, during the pandemic.

#### Data sources

We used two datasets for the present study. The first dataset included information on laboratory-confirmed cases of COVID-19 from the CDC database in Bac Ninh province (https://bacninhcdc.vn/), which provided demographic characteristics of the study population, including their age, sex, case classification (community areas –confirmed cases in the community, and quarantine areas – confirmed cases which showed up in the isolation or blockade areas), home address, and date of illness onset. The second source of information was the vaccination dataset from the CDC database in Bac Ninh province (https://bacninhcdc.vn/), which included information about the number of COVID vaccine doses administered (including first and second doses) in Bac Ninh province from 1 January to 31 December 2021. We also reviewed the timeline of local policy documents for controlling the COVID-19 pandemic in Bac Ninh province in 2020–2021.

### Data analysis

Patients’ demographic characteristics were presented as percentages and compared among the three study periods (Zero-COVID period, Transition period, and New-normal period) using Chi-square tests for categorical variables and or as means (SD), and compared using ANOVA for continuous variables. Since the results of the ANOVA test were statistically significant, we computed Tukey Honest Significant Differences for performing multiple pairwise comparisons between the means of the three study periods.

We constructed a pandemic curve that included the daily number of patients with COVID-19 (divided into community and quarantine areas), the proportion of the provincial population administered a first and second dose of the COVID vaccine, and policy actions taken by the Bac Ninh government during 2021 to prevent and control the COVID-19 pandemic.

For calculating the CCC, we only included data from confirmed cases in the community areas which were reported as the total number of daily cases of COVID-19 at the ward unit level in each of the eight districts in Bac Ninh province. We calculated three indices: the number of CCCs per ward unit (by cycle), the length of CCC per ward unit (by day), and the number of confirmed cases of COVID-19 per CCC at each ward unit (by case). The Kruskal–Wallis test was used to identify any differences in these three indices between the three study periods. All analyses were performed using the R-Program software packages (tidyverse, readxl, haven, stringi, lubridate, writexl, readxl, scales, Table 1, data.table, Rmisc, ggplot2, cowplot). Statistical significance was set at a *p-*value level of <0.05.

**Table 1.** Study population characteristics according to study time period

Characteristics n (%)	Period			*p*-value
	Zero-COVID	Transition	New-normal	
	(n = 3149)	(n = 1290)	(n = 9288)	
**Age (years)**				
Mean (SD)	30.1 (16.1)	29.2 (15.2)	28.3 (13.9)	**<0.001** ^a^
**Age group**				
< 10	293 (9.3)	128 (9.9)	763 (8.2)	**<0.001**
10–19	465 (14.8)	205 (15.9)	1266 (13.6)	
20–29	967 (30.7)	385 (29.8)	3662 (39.4)	
30–39	756 (24.0)	297 (23.0)	2156 (23.2)	
40–49	278 (8.8)	153 (11.9)	682 (7.3)	
50–59	204 (6.5)	60 (4.7)	417 (4.5)	
> = 60	186 (5.9)	62 (4.8)	341 (3.7)	
**Sex**				
Female	1711 (54.3)	723 (56.0)	4640 (50.0)	**<0.001**
**Living place**				
Bac Ninh city	1186 (37.7)	351 (27.2)	2749 (29.6)	**<0.001**
Yen Phong district	132 (4.2)	183 (14.2)	552 (5.9)	
Que Vo district	410 (13.0)	192 (14.9)	1531 (16.5)	
Tien Du district	109 (3.5)	79 (6.1)	611 (6.6)	
Tu Son town	293 (9.3)	182 (14.1)	1608 (17.3)	
Thuan Thanh district	712 (22.6)	46 (3.6)	449 (4.8)	
Gia Binh district	92 (2.9)	10 (0.8)	261 (2.8)	
Luong Tai district	28 (0.9)	104 (8.1)	204 (2.2)	
Other provinces	187 (5.9)	143 (11.1)	1323 (14.2)	
**Case classification**				
Community areas	2674 (84.9)	861 (66.7)	7064 (76.1)	**<0.001**
Quarantine areas	475 (15.1)	429 (33.3)	2224 (23.9)	

*Note*: (a) Multiple pairwise-comparison: Zero-COVID vs Transition: *p*-value = 0.20/ Transition vs New-normal: *p*-value = 0.06/ Zero-COVID vs New-normal: *p*-value < 0.001.

a ANOVA test

These are the 3 periods of the pandemic in Bac Ninh Province during the study time.

### Study ethics

All personal information was removed from the dataset before analysis. The data are considered as secondary data and Institutional Review Board review was not required. All the datasets had received permission from the Bac Ninh CDC for use and analysis.

### Patient and public involvement

None.

## Results

### Study population characteristics

There were a total of 13,727 confirmed cases of COVID-19, with complete data available during the period under study (Table 1). The number of patients was highest in those 11–40 years old, while the rate among adults 60 years and older was the lowest. The incidence rate in females was higher than in males, and almost two-thirds of the cases were diagnosed in community areas. Residents of Bac Ninh city had the highest rate of new cases of COVID-19 in the eight districts examined.

During the Zero-COVID period, the number of infected women and the mean age of patients was higher than during the other comparison periods. Bac Ninh city had the highest number of patients during the three periods, while the number of patients from community areas during the New-normal period was the greatest.

### Pandemic curves, vaccination campaign, and governmental health policy actions

#### Pandemic curves

**Figure 1.**
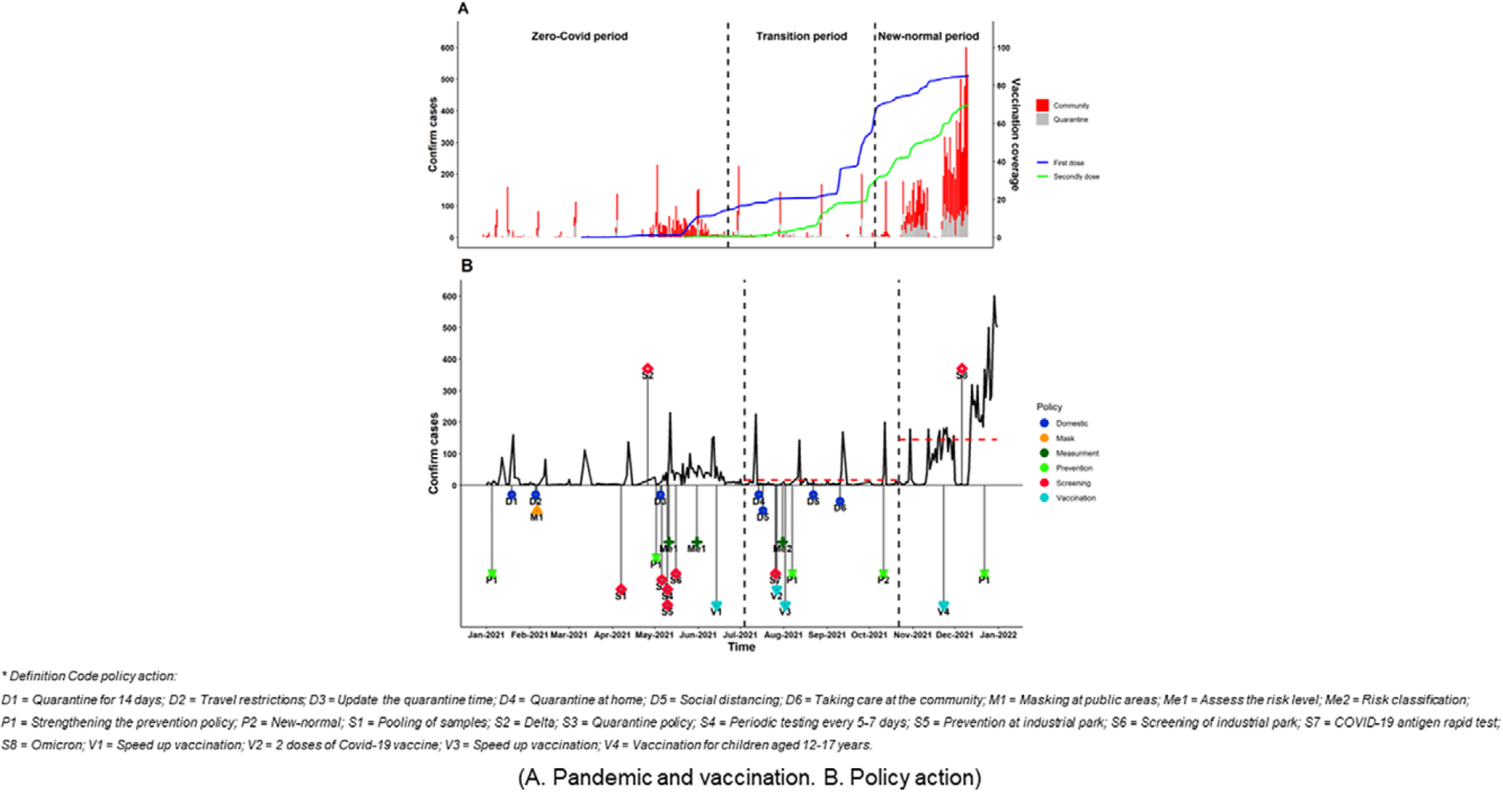
Pandemic curve with vaccination campaign and policy action at Bac Ninh province from 1 January to 31 December 2021.

During the first ‘Zero-COVID’ period (01/04–07/04/2021), between 1 January 2021 and the end of April, there were four waves of virus outbreaks with a peak of more than 150 cases occurring daily during the beginning of February 2021 (Figure 1A). During this period, almost one-half of the confirmed cases were observed in the quarantine areas. From 1 May until the end of July 2021, there were two outbreaks, with almost 250 cases occurring on a daily basis during the first week of May. In the first wave, most of the cases were from the community, whereas in the second wave, from June to July 2021, there were a large number of cases that occurred in the quarantine areas.

In the next period, namely the ‘Transition’ phase (07/05–10/22/2021), four waves of the outbreak took place between July and mid-September 2021, each lasting approximately 2 weeks. The highest daily peak of more than 200 cases was observed between the middle of July and the middle of October 2021. There were many days in Bac Ninh province with no notification of cases during this period, especially during the Transition period. The longest range of zero notification days was more than 2 weeks, from the second week of September to the end of October 2021.

Finally, in the ‘New-normal’ period (10/23–12/31/2021), after November 2021, there were three waves of outbreaks during this period; during the highest peak, there were more than 600 cases per day.

#### Vaccination campaign

The frequency of vaccination during the Zero-COVID period (01/04–07/04/2021) was the lowest, while during the ‘Transition’ phase (07/05–10/22/2021), there was a significant increase in the proportion of individuals who received their first vaccine dose between June and July 2021 (from <5% to >15%); this percentage went up to almost 25% of the population being vaccinated during August. In the middle of September 2021, the vaccination rate for those given a first vaccine dose increased rapidly and reached over 50% by the end of October 2021.

Lastly, in the ‘New-normal’ period (10/23–12/31/2021), the figure increased dramatically to nearly 85% by December 2021. Overall, the percentage of the population administered a second dose of the vaccine went up slightly after August 2021, reaching nearly 70% during the final study year.

#### Governmental policy actions

##### Domestic movement

During the initial ‘Zero-COVID’ period (01/04–07/04/2021), in January 2021, when there were fewer than 10 cases of COVID that occurred on a daily basis, government officials in Bac Ninh province began strengthening COVID-19 prevention and control measures with major focused policy actions on limiting domestic movement. This began with the need to quarantine for 14 days for high-risk cases, defined according to the National Steering Committee for COVID-19 Prevention and Control guidelines. The guidelines required each province to conduct a complete review of concentrated quarantine at the place of residence and continue monitoring for 14 days; travel restrictions were also imposed throughout the province during this period.

Next, in the Transition period, the Bac Ninh government focused on domestic actions that could be undertaken in the community, including reducing the time for being quarantined and the need for quarantining at home for the close contacts of infected persons, which began during mid-July 2021. At the beginning of July, suspected cases of COVID were encouraged to self-quarantine at home. Social distancing measures for the province were applied twice, once in the middle of July and again during August.

##### Mask mandate

In the first ‘Zero-COVID’ period (01/04–07/04/2021), residents of Bac Ninh province were asked to wear masks in public during the first week of February and continue this even after. In addition, COVID-19 samples were pooled for purposes of mass screening in the affected communities beginning in early April.

##### Measurement and classification

At the end of May 2021, the newly updated guidelines for diagnosing and treating COVID-19 caused by a new strain of Coronavirus (SARS-CoV-2), the Delta variant, were applied. During this time, a quarantine policy was implemented for people returning from pandemic areas. High-risk group residents of Bac Ninh province underwent periodic testing every 5–7 days. This group included medical staff, caregivers, and service staff (without clinical suspicion of COVID-19) in isolation facilities. It also included patients receiving inpatient treatment, individuals seeking medical examination, and those subject to home isolation (F2). Other high-risk groups consisted of people living in outbreak areas, essential workers (without clinical manifestations of COVID-19) in contact with the community, personnel at border gates and crossings, individuals working in industrial parks and companies, and those guided by the Vietnamese Department of Health. After these policies had been applied, during the Transition period, risk classification for patients was applied at the end of July and further prevention and control measures were instituted beginning during the first week of August 2021. Furthermore, patients with COVID-19 who had mild symptoms could quarantine at home and take their follow-up visits with medical staff at community facilities in the local areas.

##### Prevention and control activities

During the ‘Zero-COVID’ period, the existing prevention and control measures for COVID-19 with the Delta variant were strengthened at the beginning of May. The Bac Ninh government specifically targeted the industrial areas, combining COVID prevention and control activities with the screening of suspect cases of COVID-19 in the industrial parks. The newly updated quarantine approach was applied during the first week of May 2021, and in the middle of June, the general public was again encouraged to receive available COVID vaccines.

Most of the actions for preventing and controlling the COVID-19 pandemic, such as scheduling mass screening tests and tracking the chains of transmission in the community, were reduced during the New-normal period. After identifying the new Omicron strain in early December, the Bac Ninh government began strengthening prevention and control measures for COVID by the third week of December 2021.

##### Screening activities

During the Zero-COVID period, most policy actions screened for virus transmission in the community (Figure 1B). Beginning in April 2021, many screening actions were conducted in the community. During the Transition period, the COVID-19 antigen rapid test was used as the new standard for diagnosing confirmed cases.

##### Vaccination activities

During the last week of July, the second dose of the COVID-19 vaccine was required for all residents of Bac Ninh province. By the beginning of August 2021, the population rate of vaccine coverage markedly increased, reaching almost 70% by the end of December. At the beginning of October, the New-normal policy began to roll out in several areas. Finally, in the ‘New-normal’ period (10/23–12/31/2021), the Bac Ninh government began vaccinating children aged 12 to 17 years at the end of November.

### COVID-19 containment cycle (CCC) time

**Figure 2.**
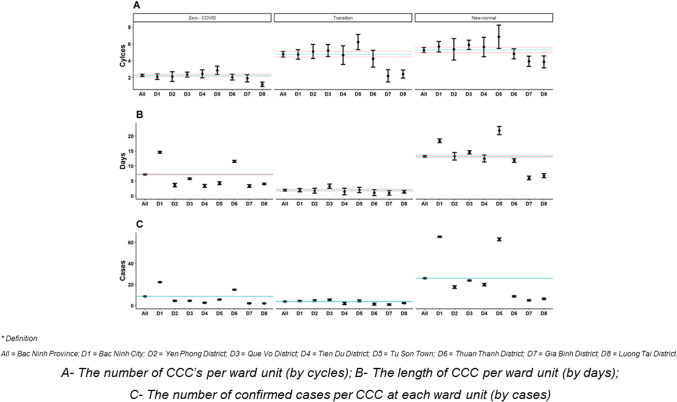
The COVID-19 containment cycle (CCC) indices at each District of Bac Ninh province.

Overall, most of the eight districts had the number and length of CCCs, and the number of confirmed cases per CCC at each ward unit was lower than the average of the overall province (Figure 2). The number of CCC cycles in the Zero-COVID period was lower than during other periods, while the lowest length of CCC and the number of confirmed cases per CCC at each ward unit was found to be during the Transition period.

In four districts (Yen Phong, Que Vo, Tien Du, and Tu Son Town), the number of CCCs per ward unit during the Zero-COVID period was lower than during the Transition and New-normal periods. In the New-normal period, the length of CCC per ward unit and the number of confirmed cases per CCC at each ward unit in each district were higher than during the other periods (Supplementary Appendix Table 2).

## Discussion

Bac Ninh province is one of Vietnam’s most industrialised provinces, and the COVID-19 outbreak devastated its manufacturing sector. The governmental response to monitoring COVID-19 hotspots helped the province to maintain the industrial ecosystem during the pandemic. Inasmuch, provincial officials’ reaction to the COVID-19 pandemic could be a lesson with broad applicability.

### Policy strategies during the Zero-COVID period

During the Zero-COVID period, in which a number of CCC measures were applied, public health efforts throughout this large province successfully controlled the spread of new cases of COVID. The Bac Ninh government implemented an elimination policy that focused on massive screening tests for the detection, isolation, and quarantining of confirmed cases of COVID and their close contacts, and the delivery of effective treatment approaches. In the industrial areas, the policy of ‘eating together, staying together, working together’ and actively screening for COVID in high-risk areas was implemented to detect infected patients [12]. Districts with large industrial parks of many workers reduced the community transmission of COVID-19 dramatically, and, by extension, the length and number of CCC’s decreased markedly during the Zero-COVID period.

In addition, during the Zero-COVID period, provincial health officials isolated the sources of viral transmission, reduced the frequency of infections, and treated individuals with COVID as promptly as possible, thereby reducing the development of cases of severe COVID. These actions led to highly effective responses since, during the beginning stages of the COVID-19 pandemic in 2021, the vaccination rate among residents of Bac Ninh province was relatively low and well below the desirable rate of vaccination to stop the spread of COVID-19 [13].

Several action measures were implemented during the Zero-COVID period, including domestic movement restrictions, mask mandates, and screening efforts. These control measures initially proved effective, as the number of confirmed cases decreased significantly, especially in districts such as Yen Phong, Que Vo, Tien Du, and Tu Son Town, where the number of CCCs per ward unit was lower compared to the subsequent periods. The focus on quarantining and screening in the community, coupled with mask-wearing, contributed to containing viral spread. Lessons learned from this period include the importance of early and stringent preventive measures, such as mask mandates and community-based screening, to quickly identify and isolate cases of possible COVID-19.

### Policy strategies during the transition period

In addition to rapidly increasing the percentage of the local population vaccinated from 25% to nearly 80% over a relatively short time, some flexible health policy measures were implemented between the Zero-COVID period and the New-normal period.

The main individual-level policies during the early stages of the Transition period were to maintain social distancing and to ‘stay where you live’, while at the district level, the focus was on re-opening businesses and reducing the number of areas that required quarantining. During this period, many areas did not record any cases of COVID for many days, and the mean length of CCC time increased from 1 to 3 days per ward unit in each of the four districts which were allowed to re-open and start the New-normal period.

To maintain the local economic system, public health and governmental officials tried isolating the industrial parks where most COVID-19 infections clustered. The industrial parks used the ‘3 staying places’ policy, where workers could rotate shifts to stay and work at their place of work over a 14-day period [12]. Furthermore, they implemented a ‘1 round 2 destinations’ policy which allowed the workers to travel only between their place of work and their home (isolation zone) during the isolation period, thereby creating a balance between controlling the pandemic and re-opening the local and regional economy [12].

During the Transition phase, the government implemented a number of measures to manage domestic actions, reduce quarantine time, and encourage home quarantining for close contacts of confirmed cases of COVID-19. While there were multiple waves of the outbreak during this period, there were extended periods with zero case notifications, particularly in Bac Ninh province. The number of CCCs per ward unit and the length of CCCs were the lowest during this period, indicating relatively successful containment efforts. However, it is also important to acknowledge that reductions in quarantine time and home quarantining could have contributed to the resurgence of cases in subsequent waves. This period highlights the need for a cautious approach when relaxing various control measures and the necessity of continued monitoring and testing, even during low transmission periods.

### Policy strategies during the New-normal period

During the New-normal period, the New-normal policy-mitigation period was applied in Bac Ninh province and throughout Vietnam [14]. Beginning in October 2021, even though the number of cases of COVID in Bac Ninh province was still high, the CCC time was quite short at just under 8 days.

The local population had a relatively high level of immunity during the New-normal period; approximately 85% of the community had received their first vaccine dose, and almost 70% had received their second dose. By the end of 2021, the majority of the population living in Bac Ninh province had received at least one dose of the COVID-19 vaccine.

The number of cases of COVID-19 surged, and the length of CCCs per ward unit increased, along with the number of confirmed cases per CCC. During this period, the reduction in preventive measures, such as mass screening and tracking transmission chains, may have contributed to the increased rates of viral transmission. After identifying the new Omicron strain in December, the subsequent strengthening of prevention and control measures highlights the necessity of prompt and decisive actions in response to emerging variants. Additionally, the successful vaccination campaign, with a significant increase in vaccine coverage, demonstrated the importance of vaccination as a crucial tool in mitigating the impact of the virus in this geographic area.

#### Lessons learned

Our findings provide valuable insights for managing similar situations in the future. Firstly, the early implementation of stringent preventive measures, such as mask mandates and community-based screening, can effectively contain the virus’s spread during the initial stages. Secondly, there is a need for cautious relaxation of measures during the transition phase, with continued monitoring and testing to identify any resurgence in viral spread. Thirdly, the importance of vaccination campaigns cannot be overstated, as higher vaccination rates were associated with better control of the virus. Lastly, adapting and responding promptly to emerging variants is crucial to strengthening preventive measures and adjusting various vaccination strategies and approaches.

#### Study strengths and limitations

The strengths of our study are that we were able to combine COVID-19 pandemic data with the governmental policy timeline to describe the effects of different policy actions in containing this pandemic. There were some limitations to our study, however, that need to be kept in mind in the interpretation of our study results. We did not have the transmission data of COVID-19 from patient to patient, so we could not examine how the policy actions may have stopped the transmission chains of the COVID-19 pandemic throughout all three periods under study.

## Conclusions

In conclusion, the present study sheds light on the successes and failures of different action measures during the Zero-COVID period, the Transition phase, and the New-normal period in Bac Ninh province. The flexibility to apply different COVID prevention strategies in Vietnam was based on successful lessons learned from many countries, including Korea, Australia, Singapore, and New Zealand. Our study has provided information on policy actions to prevent and control the COVID-19 pandemic at the provincial level by sharing experiences from one large typical province in Vietnam. The implementation of various control measures, such as mask mandates, community-based screening, and vaccination campaigns, contributed to successful community containment of COVID-19 during certain periods. However, the relaxation of measures and the reduction in preventive actions led to increased transmission rates in other periods. The lessons from this research provide valuable insights for future pandemic management, emphasising the importance of early and stringent preventive measures, cautious relaxation of different control measures, and prompt responses to emerging viral variants.

## Supporting information

Toan et al. supplementary material Toan et al. supplementary material

## Data Availability

Data are not publicly available.

## Supplementary material

The supplementary material for this article can be found at http://doi.org/10.1017/S0950268823001048.
