# Shift from a Zero-COVID strategy to a New-normal strategy for controlling SARS-COV-2 infections in Vietnam – CORRIGENDUM

**DOI:** 10.1017/S0950268826101617

**Published:** 2026-05-29

**Authors:** Do Thi Thanh Toan, Thanh Hai Pham, Khanh Cong Nguyen, Quang Thai Pham, Duc Doanh Ha, Hoa L. Nguyen, Robert J. Goldberg, Loc Quang Pham, Giang Minh Le, Tu Khac Nguyen, Van Khanh Tran, Van Thanh Ta

**Affiliations:** 1 School of Preventive Medicine and Public Health, Hanoi Medical University, Hanoi, Vietnam; 2 National Institute of Hygiene and Epidemiology, Hanoi, Vietnam; 3 Department of Population and Quantitative Health Sciences, University of Massachusetts Chan Medical School, Worcester, Massachusetts, USA; 4 Bac Ninh Center for Disease Control and Prevention, Bac Ninh, Vietnam; 5 Hanoi Medical University, Hanoi, Vietnam

An author’s name was incorrectly spelled in the article as it was originally published. This has been corrected from Quoc Doanh Ha to Duc Doanh Ha in the HTML and PDF versions of the article. The authors apologise for the error.
