# Supplementary material for: Shift from a Zero-COVID strategy to a New-normal strategy for controlling SARS-COV-2 infections in Vietnam
Source: Epidemiol Infect. 2023 Jul 4;151:e117. doi: 10.1017/S0950268823001048 (PMC10644050; doi:10.1017/S0950268823001048)
Supplement: Toan et al. supplementary material [file S0950268823001048sup001.docx]

#### Appendix Table 1. Definition of the health policy disease control strategies

| **ID** | **Group actions** | **Period** | | |
| --- | --- | --- | --- | --- |
|  |  | **Zero-COVID** | **Transition** | **New-normal** |
| **1** | **Domestic movement** | Extremely high levels of social distancing restrictions are required. | Reduce the levels of social distancing restrictions. Only approach the social distance at high-risk areas. | No need the social distancing restrictions. |
| **2** | **Mask mandate** | Required the mask at community areas. | Required the mask at community areas. | Reduce the mask at community areas. Require mask at large group areas |
| **3** | **Measurement and Classification** | Classify and quarantine all the suspected cases who had history contact with confirmed cases. Isolating all the suspected and confirmed cases at specific quarantine areas. | Level the risk level of subjects and conduct home isolation with low level suspect subject. | Home isolation and home treatment with suspected and confirmed cases. |
| **4** | **Prevention and controlling** | Achieving no community transmission. All public health control measures were applied to eliminate the occurrence of SARS-Cov-2 in the community.  Quarantine at specific isolation areas for both cases and close contacts. | Accepting community transmission with low level. If the community cases increase, high level of public health control measure will be applied.  Self -Quarantine at home for both cases and close contacts | Accepting community transmission. Only conducting the high level of public health control measure if required.  Self -Quarantine at home for confirmed cases |
| **5** | **Screening** | Mass test and tracking the transmission chains of suspected cases. PCR test was used as standard for confirmed cases. Test mandate both cases and close contacts | Reducing the tracking the transmission chains. Testing weekly at high level areas Test mandate both cases and close contacts | Not required tracking the transmission chains. Rapid test be used as the standard for confirmed cases. |
| **6** | **Vaccination** | Allocate vaccines to priority groups. | Vaccination for all people aged 18 years and above. | Vaccination for all people aged 12 years and above. |

#### Appendix Table 2. COVID-19 Containment cycle (CCC) indices at each District of Bac Ninh Province

| **A- Bac Ninh City** | **Total** | **Period** | | | **p-value *** | **Multiple pairwise-comparison** | | |
| --- | --- | --- | --- | --- | --- | --- | --- | --- |
|  |  | **Zero - COVID** | **Transition** | **New-normal** |  | **(1)**  **vs**  **(2)** | **(2)**  **vs**  **(3)** | **(1)**  **vs**  **(3)** |
|  |  | **(1)** | **(2)** | **(3)** |  | **p-value** | **p-value** | **p-value** |
|  | **(n=121)** | **(n=49)** | **(n=39)** | **(n=33)** |  |  |  |  |
| **• Number of CCC per ward unit** | | | | | | | | |
| Mean  (95%CI) | 3.9  (3.5 – 4.3) | 2.1  (1.8 - 2.4) | 4.7  (4.2 - 5.3) | 5.7  (5.0 - 6.3) | **<0.001** | **<0.001** | **0.030** | **<0.001** |
| Median  (Q1 – Q3) | 4  (2 - 5) | 2  (1 - 3) | 4  (3.5 - 6) | 6  (5 - 7) |  |  |  |  |
| **• Length of CCC per ward unit** | | | | | | | | |
| Mean  (95% CI) | 11.5  (11.1 – 11.9) | 14.6  (14.2 - 14.9) | 1.9  (1.4 - 2.5) | 18.4  (17.8 - 19.0) | **<0.001** | **<0.001** | **<0.001** | **0.020** |
| Median  (Q1 - Q3) | 2  (1 - 16) | 5  (1 - 18) | 1  (1 - 1) | 16  (6 - 19) |  |  |  |  |
| **• Number of confirm cases per CCC at each ward unit** | | | | | | | | |
| Mean  (95% CI) | 28.2  (27.8 - 28.6) | 22.2  (21.9 - 22.5) | 4.3  (3.7 - 4.9) | 65.3  (64.7 - 65.9) | **<0.001** | **0.008** | **<0.001** | **0.039** |
| Median  (Q1 - Q3) | 4  (1 - 14) | 3  (1 - 15) | 1  (1 - 8) | 11  (4 - 23) |  |  |  |  |

** Kruskal-Wallis test*

####

#### Appendix Table 2 (continued). The CCC indices at each District of Bac Ninh Province

| **B- Yen Phong District** | **Total** | **Period** | | | **p-value *** | **Multiple pairwise-comparison** | | |
| --- | --- | --- | --- | --- | --- | --- | --- | --- |
|  |  | **Zero - COVID** | **Transition** | **New-normal** |  | **(1)**  **vs**  **(2)** | **(2)**  **vs**  **(3)** | **(1)**  **vs**  **(3)** |
|  |  | **(1)** | **(2)** | **(3)** |  | **p-value** | **p-value** | **p-value** |
|  | **(n=77)** | **(n=26)** | **(n=28)** | **(n=23)** |  |  |  |  |
| **• Number of CCC per ward unit** | | | | | | | | |
| Mean  (95% CI) | 4.2 (3.6 - 4.8) | 2.1  (1.5 - 2.7) | 5.1  (4.3 - 5.9) | 5.4  (4.1 - 6.6) | **<0.001** | **<0.001** | 1 | **<0.001** |
| Median  (Q1 - Q3) | 4  (2 - 6) | 1.5  (1 – 3) | 5  (3 – 6) | 5  (3 - 7) |  |  |  |  |
| **• Length of CCC per ward unit** | | | | | | | | |
| Mean  (95% CI) | 5.8  (5.2 - 6.4) | 3.6  (3.0 - 4.2) | 1.8  (0.9 - 2.6) | 13.2  (11.9 - 14.5) | **<0.001** | 0.12 | **<0.001** | **0.001** |
| Median  (Q1 - Q3) | 1  (1 - 11) | 1  (1 – 2) | 1  (1 - 1) | 14  (1.5 – 17.5) |  |  |  |  |
| **• Number of confirm cases per CCC at each ward unit** | | | | | | | | |
| Mean  (95% CI) | 8.5  (7.9 - 9.1) | 4.5  (4.0 - 5.1) | 4.8  (3.9 - 5.6) | 17.5  (16.2 - 18.8) | **0.009** | 0.69 | **0.016** | **0.016** |
| Median  (Q1 - Q3) | 3  (1 - 7) | 2.5  (1 - 5) | 2.5  (1 - 5) | 7  (3 - 24) |  |  |  |  |

** Kruskal-Wallis test*

####

#### Appendix Table 2 (continued). The CCC indices at each District of Bac Ninh Province

| **C- Que Vo District** | **Total** | **Period** | | | **p-value *** | **Multiple pairwise-comparison** | | |
| --- | --- | --- | --- | --- | --- | --- | --- | --- |
|  |  | **Zero - COVID** | **Transition** | **New-normal** |  | **(1)**  **vs**  **(2)** | **(2)**  **vs**  **(3)** | **(1)**  **vs**  **(3)** |
|  |  | **(1)** | **(2)** | **(3)** |  | **p-value** | **p-value** | **p-value** |
|  | **(n=140)** | **(n=65)** | **(n=29)** | **(n=46)** |  |  |  |  |
| **• Number of CCC per ward unit** | | | | | | | | |
| Mean  (95% CI) | 4.1  (3.7 - 4.5) | 2.4  (2.1 - 2.7) | 5.2  (4.5 - 5.9) | 5.9  (5.3 - 6.4) | **<0.001** | **<0.001** | 0.14 | **<0.001** |
| Median  (Q1 - Q3) | 4  (2 - 6) | 2  (1 - 3) | 5  (4 - 6) | 6  (4.25 - 7) |  |  |  |  |
| **• Length of CCC per ward unit** | | | | | | | | |
| Mean  (95% CI) | 8.1  (7.7 - 8.5) | 5.8  (5.5 - 6.0) | 3.2  (2.5 – 4.0) | 14.5  (14.0 - 15.1) | **<0.001** | 0.50 | **<0.001** | **<0.001** |
| Median  (Q1 - Q3) | 1  (1 - 12) | 1  (1 - 8) | 1  (1 - 2) | 13.5  (5 – 17) |  |  |  |  |
| **• Number of confirm cases per CCC at each ward unit** | | | | | | | | |
| Mean  (95% CI) | 11.1  (10.7 - 11.4) | 4.6  (4.3 - 4.9) | 5.6  (4.9 - 6.3) | 23.8  (23.3 - 24.4) | **<0.001** | 0.75 | **<0.001** | **<0.001** |
| Median  (Q1 - Q3) | 3  (1 - 7) | 1  (1 - 5) | 2  (1 - 5) | 6  (3 - 10) |  |  |  |  |

** Kruskal-Wallis test*

####

#### Appendix Table 2 (continued). The CCC indices at each District of Bac Ninh Province

| **D- Tien Du District** | **Total** | **Period** | | | **p-value *** | **Multiple pairwise-comparison** | | |
| --- | --- | --- | --- | --- | --- | --- | --- | --- |
|  |  | **Zero - COVID** | **Transition** | **New-normal** |  | **(1)**  **vs**  **(2)** | **(2)**  **vs**  **(3)** | **(1)**  **vs**  **(3)** |
|  |  | **(1)** | **(2)** | **(3)** |  | **p-value** | **p-value** | **p-value** |
|  | **(n=79)** | **(n=34)** | **(n=21)** | **(n=24)** |  |  |  |  |
| **• Number of CCC per ward unit** | | | | | | | | |
| Mean  (95% CI) | 4  (3.4 - 4.6) | 2.4  (2.0 - 2.9) | 4.7  (3.6 - 5.8) | 5.6  (4.5 - 6.8) | **<0.001** | **0.002** | 0.25 | **<0.001** |
| Median  (Q1 - Q3) | 3  (2 - 6) | 2  (1 - 3) | 5  (4 - 6) | 5.5  (3 - 7) |  |  |  |  |
| **• Length of CCC per ward unit** | | | | | | | | |
| Mean  (95% CI) | 5.6  (5.0 - 6.2) | 3.4  (2.9 - 3.8) | 1.5  (0.4 - 2.6) | 12.5  (11.3 - 13.6) | **<0.001** | 0.32 | **<0.001** | **<0.001** |
| Median  (Q1 - Q3) | 1  (1 – 6.5) | 1  (1 - 1) | 1  (1 - 1) | 14.5  (1 – 17.5) |  |  |  |  |
| **• Number of confirm cases per CCC at each ward unit** | | | | | | | | |
| Mean  (95% CI) | 7.7  (7.1 - 8.3) | 2.7  (2.3 -3.2) | 2  (0.8 - 3.1) | 19.8  (18.7 - 21.0) | **<0.001** | 0.53 | **0.001** | **0.001** |
| Median  (Q1 - Q3) | 2  (1 - 4) | 1.5  (1 - 4) | 1  (1 - 3) | 10  (2 - 19) |  |  |  |  |

** Kruskal-Wallis test*

####

#### Appendix Table 2 (continued). The CCC index at each District of Bac Ninh Province

| **E- Tu Son Town** | **Total** | **Period** | | | **p-value *** | **Multiple pairwise-comparison** | | |
| --- | --- | --- | --- | --- | --- | --- | --- | --- |
|  |  | **Zero - COVID** | **Transition** | **New-normal** |  | **(1)**  **vs**  **(2)** | **(2)**  **vs**  **(3)** | **(1)**  **vs**  **(3)** |
|  |  | **(1)** | **(2)** | **(3)** |  | **p-value** | **p-value** | **p-value** |
|  | **(n=89)** | **(n=42)** | **(n=28)** | **(n=19)** |  |  |  |  |
| **• Number of CCC per ward unit** | | | | | | | | |
| Mean  (95% CI) | 4.8  (4.2 - 5.4) | 2.9  (2.4 - 3.4) | 6.2  (5.3 - 7.1) | 6.8  (5.5 - 8.2) | **<0.001** | **<0.001** | 0.51 | **<0.001** |
| Median  (Q1 - Q3) | 4  (2 - 7) | 3  (1 - 4) | 7  (4 - 8) | 6  (4.5 - 9) |  |  |  |  |
| **• Length of CCC per ward unit** | | | | | | | | |
| Mean  (95% CI) | 7.3  (6.7 - 7.9) | 4.2  (3.7 - 4.7) | 2  (1.1 - 2.9) | 21.8  (20.4 - 23.2) | **<0.001** | 0.11 | **<0.001** | **<0.001** |
| Median  (Q1 - Q3) | 1  (1 - 11) | 1  (1 - 5) | 1  (1 - 1) | 17  (7 - 28) |  |  |  |  |
| **• Number of confirm cases per CCC at each ward unit** | | | | | | | | |
| Mean  (95% CI) | 17.5  (16.9 - 18.1) | 5.7  (5.2 - 6.2) | 4.5  (3.6 - 5.4) | 62.8  (61.5- 64.2) | **<0.001** | 0.49 | **<0.001** | **<0.001** |
| Median  (Q1 - Q3) | 4  (1 - 10) | 3  (1 - 7) | 2.5  (1 - 6) | 33  (6 – 95.5) |  |  |  |  |

** Kruskal-Wallis test*

####

#### Appendix Table 2 (continued). The CCC index at each District of Bac Ninh Province

| **F- Thuan Thanh District** | **Total** | **Period** | | | **p-value *** | **Multiple pairwise-comparison** | | |  |
| --- | --- | --- | --- | --- | --- | --- | --- | --- | --- |
|  |  | **Zero - COVID** | **Transition** | **New-normal** |  | **(1)**  **vs**  **(2)** | **(2)**  **vs**  **(3)** | **(1)**  **vs**  **(3)** | |
|  |  | **(1)** | **(2)** | **(3)** |  | **p-value** | **p-value** | **p-value** | |
|  | **(n=92)** | **(n=45)** | **(n=13)** | **(n=34)** |  |  |  |  |  |
| **• Number of CCC per ward unit** | | | | | | | | | |
| Mean  (95% CI) | 3.4  (3.0 - 3.8) | 2.0 (1.7 - 2.4) | 4.2  (3.2 - 5.2) | 4.8  (4.2 - 5.4) | **<0.001** | **<0.001** | 0.21 | **<0.001** | |
| Median  (Q1 - Q3) | 3  (2 - 5) | 2  (1 - 3) | 3  (3 - 5) | 4.5  (4 - 5) |  |  |  |  |  |
| **• Length of CCC per ward unit** | | | | | | | | | |
| Mean  (95% CI) | 10.2  (9.7 - 10.6) | 11.5  (11.2 - 11.9) | 1.1  (0.1- 2.1) | 11.8  (11.2 - 12.4) | **<0.001** | **0.006** | **<0.001** | 0.25 | |
| Median  (Q1 - Q3) | 4  (1 - 16) | 3  (1 - 19) | 1  (1 - 1) | 12  (3 - 17) |  |  |  |  |  |
| **• Number of confirm cases per CCC at each ward unit** | | | | | | | | | |
| Mean  (95% CI) | 10.8  (10.4 - 11.2) | 15.0  (14.7 - 15.4) | 1.4  (0.4 - 2.4) | 8.8  (8.2 - 9.4) | **<0.001** | **0.003** | **<0.001** | **0.043** | |
| Median  (Q1 - Q3) | 3  (1 - 9) | 3  (1 - 6) | 1  (1 - 1) | 6.5  (3 - 11) |  |  |  |  |  |

** Kruskal-Wallis test*

#### Appendix Table 2 (continued). The CCC indices at each District of Bac Ninh Province

| **G- Gia Binh District** | **Total** | **Period** | | | **p-value *** | **Multiple pairwise-comparison** | | |
| --- | --- | --- | --- | --- | --- | --- | --- | --- |
|  |  | **Zero - COVID** | **Transition** | **New-normal** |  | **(1)**  **vs**  **(2)** | **(2)**  **vs**  **(3)** | **(1)**  **vs**  **(3)** |
|  |  | **(1)** | **(2)** | **(3)** |  | **p-value** | **p-value** | **p-value** |
|  | **(n=56)** | **(n=24)** | **(n=5)** | **(n=27)** |  |  |  |  |
| **• Number of CCC per ward unit** | | | | | | | | |
| Mean  (95% CI) | 2.9  (2.5 - 3.3) | 1.9  (1.5 - 2.4) | 2.2  (1.5 - 2.9) | 3.9  (3.3 - 4.5) | **<0.001** | 0.39 | **0.028** | **<0.001** |
| Median  (Q1 - Q3) | 3  (2 - 4) | 2  (1 - 2) | 2  (2 - 3) | 4  (3 - 5) |  |  |  |  |
| **• Length of CCC per ward unit** | | | | | | | | |
| Mean  (95% CI) | 4.4  (4.0 - 4.8) | 3.3  (2.8 - 3.7) | 1.0 (0.3 - 1.7) | 6.0 (5.4 - 6.6) | **0.006** | 0.29 | **0.032** | **0.031** |
| Median  (Q1 - Q3) | 1  (1 - 6) | 1  (1 - 1) | 1  (1 - 1) | 4  (1 - 10) |  |  |  |  |
| **• Number of confirm cases per CCC at each ward unit** | | | | | | | | |
| Mean  (95% CI) | 3.4  (3.0 - 3.8) | 2.1  (1.7 - 2. 6) | 1.0 (0.3 - 1.7) | 5.0  (4.4 - 5.6) | **0.024** | 0.12 | **0.045** | 0.12 |
| Median  (Q1 - Q3) | 1  (1 - 3) | 1  (1 - 3) | 1  (1 - 1) | 2  (1 – 3.5) |  |  |  |  |

** Kruskal-Wallis test*

####

#### Appendix Table 2 (continued). The CCC indices at each District of Bac Ninh Province

| **H- Luong Tai District** | **Total** | **Period** | | | **p-value *** | **Multiple pairwise-comparison** | | |
| --- | --- | --- | --- | --- | --- | --- | --- | --- |
|  |  | **Zero - COVID** | **Transition** | **New-normal** |  | **(1)**  **vs**  **(2)** | **(2)**  **vs**  **(3)** | **(1)**  **vs**  **(3)** |
|  |  | **(1)** | **(2)** | **(3)** |  | **p-value** | **p-value** | **p-value** |
|  | **(n=56)** | **(n=24)** | **(n=5)** | **(n=27)** |  |  |  |  |
| **• Number of CCC per ward unit** | | | | | | | | |
| Mean  (95% CI) | 2.8  (2.3 - 3.3) | 1.2  (0.9 - 1.5) | 2.4  (1.9 - 2.9) | 3.9  (3.2 - 4.6) | **<0.001** | **0.002** | **0.005** | **<0.001** |
| Median  (Q1 - Q3) | 2.5  (1 - 4) | 1  (1 - 1) | 2  (2 - 3) | 4  (3 - 5) |  |  |  |  |
| **• Length of CCC per ward unit** | | | | | | | | |
| Mean  (95% CI) | 4.3  (3.8 - 4.8) | 4  (3.7 - 4.3) | 1.4  (0.9 - 1.9) | 6.8  (6.1 - 7.5) | **0.003** | 0.54 | **0.004** | 0.10 |
| Median  (Q1 - Q3) | 1  (1 - 5) | 1  (1 - 1) | 1  (1 - 1) | 4  (1 - 13) |  |  |  |  |
| **• Number of confirm cases per CCC at each ward unit** | | | | | | | | |
| Mean  (95% CI) | 4.1  (3.6 - 4.6) | 2.1  (1.8 - 2.4) | 2.4  (1.9 - 2.9) | 6.4  (5.7 - 7.1) | **0.047** | 0.79 | 0.08 | 0.08 |
| Median  (Q1 - Q3) | 1.5  (1 - 4) | 1  (1 - 2) | 1  (1 - 3) | 3  (1 - 7) |  |  |  |  |

** Kruskal-Wallis test*

####

#### Appendix Table 2 (continued). The CCC indices at each District of Bac Ninh Province

| **Bac Ninh Province** | **Total** | **Period** | | | **p-value *** | **Multiple pairwise-comparison** | | |
| --- | --- | --- | --- | --- | --- | --- | --- | --- |
|  |  | **Zero - COVID** | **Transition** | **New-normal** |  | **(1)**  **vs**  **(2)** | **(2)**  **vs**  **(3)** | **(1)**  **vs**  **(3)** |
|  |  | **(1)** | **(2)** | **(3)** |  | **p-value** | **p-value** | **p-value** |
|  | **(n=56)** | **(n=24)** | **(n=5)** | **(n=27)** |  |  |  |  |
| **• Number of CCC per ward unit** | | | | | | | | |
| Mean  (95% CI) | 3.9  (3.7 - 4.0) | 2.3  (2.1 - 2.4) | 4.8  (4.4 - 5.1) | 5.3  (5.0 - 5.6) | **<0.001** | **<0.001** | **0.041** | **<0.001** |
| Median  (Q1 - Q3) | 3  (2 - 5) | 2  (1 - 3) | 4.5  (3 - 6) | 5  (3.5 - 7) |  |  |  |  |
| **• Length of CCC per ward unit** | | | | | | | | |
| Mean  (95% CI) | 7.8  (7.6 - 7.9) | 7.2  (7.0 - 7.3) | 1.9  (1.6 - 2.3) | 13.2  (12.9 - 13.5) | **<0.001** | **<0.001** | **<0.001** | **<0.001** |
| Median  (Q1 - Q3) | 1  (1 - 12) | 1  (1 - 10) | 1  (1 - 1) | 12  (2 - 17) |  |  |  |  |
| **• Number of confirm cases per CCC at each ward unit** | | | | | | | | |
| Mean  (95% CI) | 13.1  (12.9 - 13.2) | 8.8  (8.6 - 8.9) | 3.9  (3.5 - 4.2) | 26.0  (25.6 - 26.3) | **<0.001** | **0.017** | **<0.001** | **<0.001** |
| Median  (Q1 - Q3) | 3  (1 - 8) | 2  (1 - 5) | 1  (1 - 4) | 6  (2 - 16) |  |  |  |  |

** Kruskal-Wallis test*
